# Development of Electrohysterogram Recording System for Monitoring Uterine Contraction

**DOI:** 10.1155/2019/4230157

**Published:** 2019-07-01

**Authors:** Dongmei Hao, Yang An, Xiangyun Qiao, Qian Qiu, Xiya Zhou, Jin Peng

**Affiliations:** ^1^College of Life Science and Bioengineering, Beijing University of Technology, Intelligent Physiological Measurement and Clinical Translation, Beijing International Base for Scientific and Technological Cooperation, Beijing 100024, China; ^2^Department of Obstetrics, Peking Union Medical College Hospital, Beijing 100730, China

## Abstract

Uterine contraction (UC) is an important clinical indictor for monitoring uterine activity. The purpose of this study is to develop a portable electrohysterogram (EHG) recording system (called PregCare) for monitoring UCs with EHG signals. The PregCare consisted of sensors, a signal acquisition device, and a computer with application software. Eight-channel EHG signals, the tocodynamometry (TOCO) signal, and maternal perception were recorded simultaneously by the signal acquisition device controlled by the computer via Bluetooth. PregCare was firstly evaluated by a signal simulator. Its relative error (RE) and coefficient of variation (CV) were calculated, and its agreement with the commercial instrument PowerLab was assessed by Bland–Altman plots. After that, PregCare was applied to 20 pregnant women in a hospital to record their EHG signals. These EHG signals were preprocessed and segmented into UCs and non-UCs. Then, the EHG features corresponding to UCs and non-UCs were extracted, respectively, including power spectral density (PSD), root mean square (RMS), peak frequency (PF), median frequency (MDF), and sample entropy (SamEn). One-way ANOVA was employed to assess the difference between UCs and non-UCs. The results show that RE and CV were less than 8% and 0.03%, respectively, which indicated the high accuracy and repeatability of PregCare. The small differences of mean and standard deviation indicated the high agreement between PregCare and PowerLab. Besides, the PSD of UCs was much larger than non-UCs between 0 and 0.7 Hz. RMS of UCs was significantly larger than non-UCs (*p* < 0.05). PF and SamEn of UCs were significantly smaller than non-UCs (*p* < 0.05). In conclusion, the developed EHG recording system was able to record EHG signals reliably. It has the advantages of portability, low power consumption, and wireless transmission, which can be used for long-term monitoring of UCs and prediction of the preterm delivery.

## 1. Introduction

Uterine contraction (UC) is an important diagnostic tool used during both pregnancy and labor. It reflects the adequacy of uterine activity and is essential to assess progress of labor. Clinical available methods of UC monitoring include manual palpation, external tocodynamometry (TOCO), and internal uterine pressure catheter (IUPC). Palpation is inexpensive and harmless but requires the constant bedside presence of a trained observer. TOCO measures UCs by means of a strain gauge tied to the abdomen of pregnant women with a belt. It detects the changes of the abdominal contour caused by UCs and converts the strain to electrical signals. TOCO is noninvasive, but its recording quality is influenced by maternal movements and amount of subcutaneous fat [1, 2]. IUPC employs a pressure transducer inserted into the uterine cavity, which directly measures the intrauterine pressure changes created by UCs. However, it is limited by its invasiveness and suffers from ruptured membranes and infection [3, 4].

Electrohysterogram (EHG), which is recorded noninvasively by electrodes on the abdominal surface of pregnant women, is representative of the electrical activity of the uterine muscle. Uterine electrical activity is the result of the depolarization and repolarization of thousands of myometrial smooth muscle cells [5]. Uterine contractility is the direct consequence of the underlying electrical activity in the myometrial cells. As the trigger of the contraction, EHG is more suitable for detection of UCs than by TOCO. EHG has been extensively investigated to identify UCs and predict preterm delivery. Many studies have proposed various signal processing techniques to extract linear, nonlinear, and propagation features of EHG to distinguish UCs from term and preterm delivery [6–8]. However, no consistent results have been obtained. In addition to different subjects and the complex EHG signals, various EHG recording devices applied in the previous studies may be one of the reasons for the inconsistence.

The measurements in the Icelandic 16-electrode EHG database were performed using a sixteen channel multipurpose physiological signal recorder (Embla A10, Medcare, Broomfield, CO, USA), most commonly used for investigating sleep disorders [9]. St Joseph's Hospital and Medical Center applied a custom-built EHG patient-monitoring system to detect contraction. TOCO was used as reference simultaneously, and the time instants at which the patient felt a UC were annotated [10]. To identify and track UCs during labor, a six-channel electromyography device that based on the LabJack U3 series DAQ was used to collect data. And the data were logged and viewed using a combination of LJStream UD20 [11]. To study the signal quality obtained from different electrodes in EHG recordings, EHG signals were amplified and filtered by the commercial biosignal amplifiers Biopac ECG100C (Biopac Systems Inc., USA). TOCO signal was recorded by Corometrics 250cx Series monitor (GE Healthcare, General Electric Company, USA) [12]. With regard to the EHG conduction velocity estimation for both the speed and direction of single spike propagation, EHG was recorded using a patch containing 4 monopolar electrodes in a diamond-shaped pattern and a ground electrode. EHG was amplified by the Porti amplifier (Twente Medical Systems International B.V.) without any visible reading of the measurement, and the data were stored directly on its flash memory [13]. A 8 × 8 electrode grid was used to investigate the EHG conduction velocity for detecting imminent delivery. These EHG signals were recorded using a Refa multichannel amplifier (TMS International, Enschede, The Netherlands) [14, 15]. In the evaluation of EHG in predicting preterm birth, a custom created system (Neuron-Spectrum 5, Neurosoft Ltd, Russia) was used. The system allowed 8-channel signal registration in 8 different points of the abdominal wall over the pregnant uterus [16]. Besides, DAS-8/PGA (Keithley Metrabyte Co., Taunton, MA) has been used to acquire electromyographic activity, and at the same time, cardiotocographic monitoring (Hewlett Packard 8030; Hewlett-Packard Co., Cupertino, CA) was applied to predict preterm birth [17].

It could be seen that there were few devices specially developed for EHG signal acquisition. Most of EHG signals were recorded by general physiological signal acquisition instruments, which were heavy and not portable for pregnant women. In particularly, EHG, TOCO, and maternal perception, which represent electrical propagation, mechanical transmission, and nerve conduction, respectively, were not recorded simultaneously by the same device, thus influencing the time comparison between these signals.

The purpose of this study is to develop a low-cost portable EHG recording system, which can be used for long-term monitoring of UCs with EHG signals. At present, the recording system will be specially developed for scientific research on UCs with the simultaneous recording of EHG, TOCO signal, and maternal perception. The recording system will be evaluated by a signal simulator and on pregnant women clinically. The EHG signals collected from the pregnant women will be analysed to demonstrate the applicability for monitoring UCs. The system can be applied to clinical practice after optimization.

## 2. EHG Recording System Development

**Figure 1:**
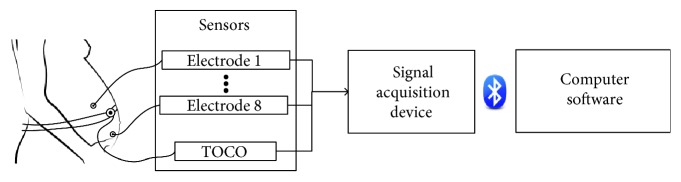
Block diagram of PregCare.

As shown in Figure 1, the developed EHG recording system (called PregCare) consisted of sensors, a signal acquisition device, and a computer with application software. The sensors included EHG electrodes and TOCO transducer. The signal acquisition device collected EHG and TOCO signals synchronously by EHG electrodes and TOCO transducer and wirelessly transmitted data to the computer. The EHG and TOCO signals were displayed on the computer screen and stored in the computer.

### 2.1. Electrode Configuration

**Figure 2:**
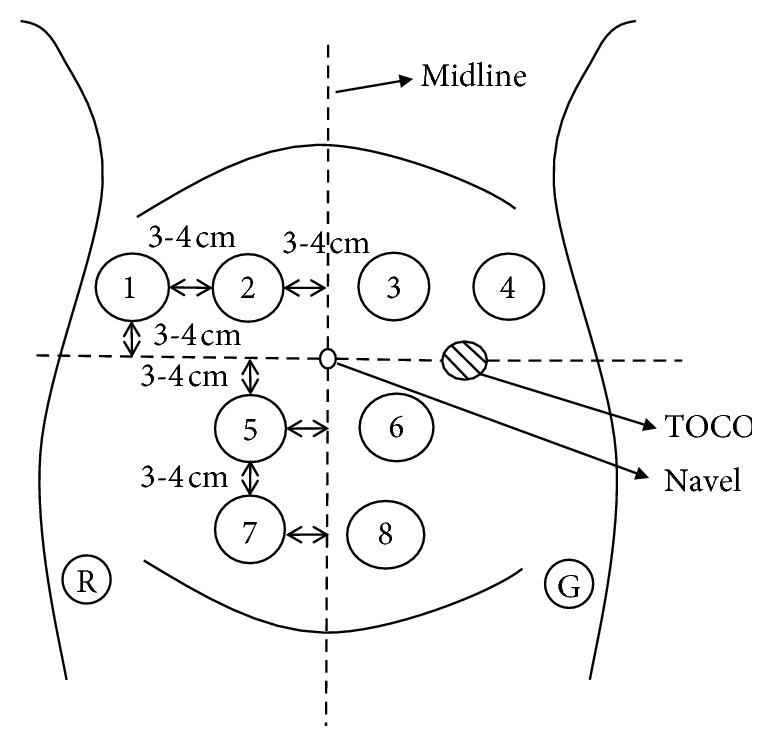
Electrode configuration. Electrode 1—position so that the edge of electrode was 6–8 cm on the left of the navel and 3-4 cm above the navel; Electrode 2—position so that the edge of electrode was 3-4 cm on the left of the navel and 3-4 cm above the navel; Electrode 5—position so that the edge of electrode was 3-4 cm left of the navel and 3-4 cm below the navel; Electrode 7—position so that the edge of electrode was 3-4 cm left of the navel and 6–8 cm below the navel; Electrodes 4 and 1, Electrodes 3 and 2, Electrodes 6 and 5, and Electrodes 8 and 7 are symmetrical about the midline; reference: Electrode R—on the left of ilium; ground: Electrode G—on the right of ilium.

The disposable electrocardiograph (ECG) electrodes (L-00-S AMBU Denmark) were applied to collect EHG signals. Considering the inverted cone of a uterus, the electrode configuration is shown as Figure 2.

### 2.2. Signal Acquisition Device

#### 2.2.1. Components of the Signal Acquisition Device

**Figure 3:**
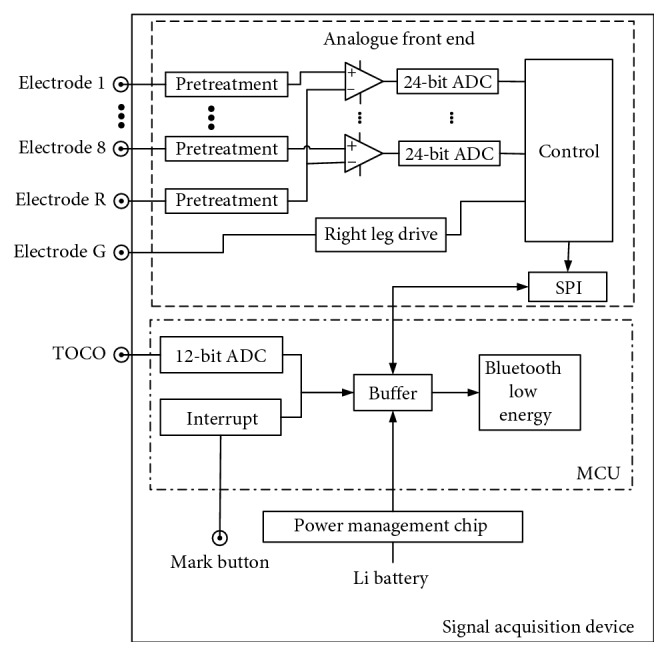
Signal acquisition device.

As shown in Figure 3, the signal acquisition device consisted of an analogue front end (AFE), a microcontroller unit (MCU), a power management chip, and a mark button. The 8-electrode EHG signals collected from the maternal abdomen surface were differentially amplified and digitized by 24 bit analogue-to-digital converters (ADC) controlled by the MCU through the serial peripheral interface (SPI). TOCO signals were digitized by a 12-bit ADC within the MCU. The mark button was pressed to record the current time when pregnant women feel UCs. The EHG signals, TOCO signals, and marks time were packaged in a buffer and sent to the computer via an integrated Bluetooth Low Energy (BLE) connection with a 2.4 GHz antenna. The device was powered by a 3.7 V lithium-polymer (Li) battery, which was monitored by the MCU.

#### 2.2.2. Firmware Embedded in the Signal Acquisition Device

**Figure 4:**
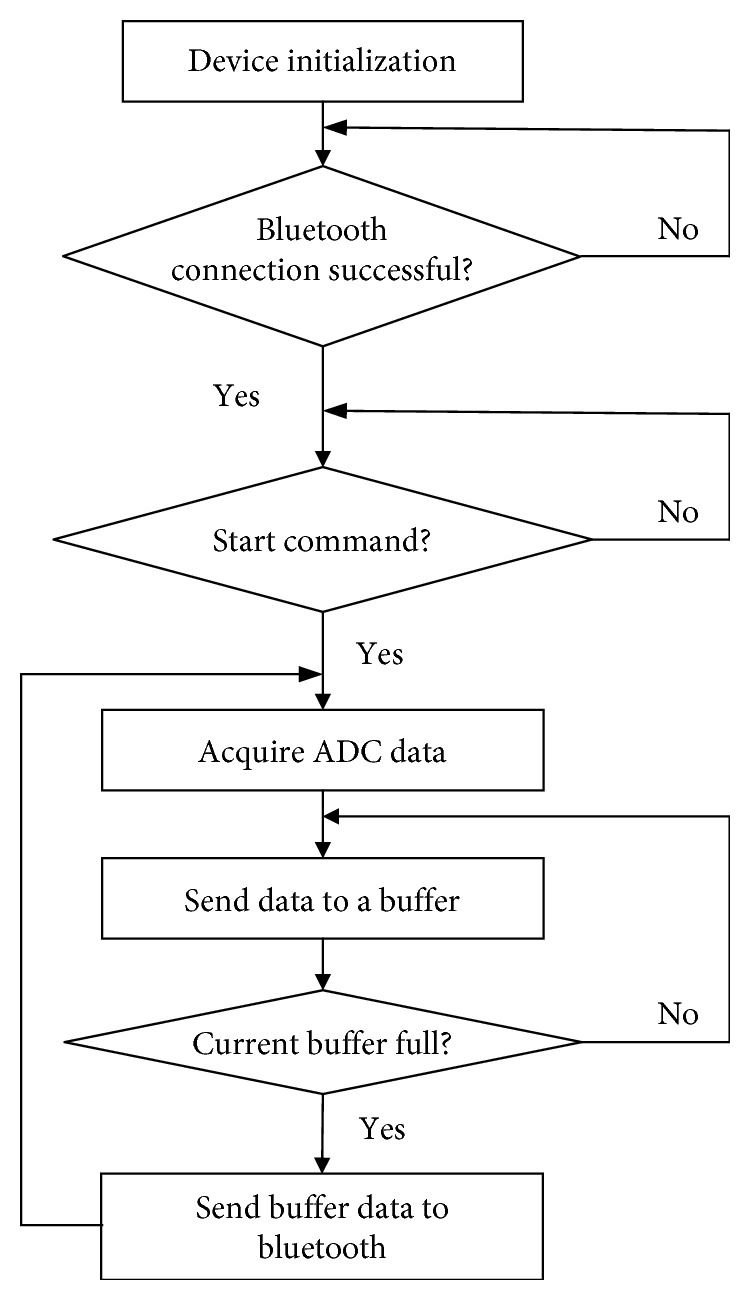
Firmware flow chart.

The firmware was responsible for collecting TOCO and EHG signals simultaneously, recording maternal perception, and packaging and sending these data to the computer via BLE.  Figure 4 shows the flow chart of the firmware. Firstly, AFE and MCU were initialized, including the system clock, timer, registers, and ADC. After the Bluetooth connection succeeded and received the start command from the computer, ADC data were acquired and sent to a buffer. When the buffer was full, the buffer data were packaged and transmitted to the computer via Bluetooth.

### 2.3. Software in the Computer

The software in the computer was developed with Visual Studio 2013. It was responsible for starting and stopping the signal acquisition device, displaying the EHG, TOCO signals, and UC marks on the computer screen, and saving data. The information about the age, height, weight, and gestational week of pregnant women was recorded.

## 3. Evaluation of the EHG Recording System

### 3.1. Evaluation of PregCare with Signal Simulator

#### 3.1.1. Experiment Design

Experiments were designed to evaluate the performance of PregCare. Because of the lack of the EHG signal simulator, the ECG signal generated by a vital signs simulator (ProSim™, Fluke Co, USA) was adopted to test the output of PregCare. Similar to EHG signals, ECG signals with the amplitude of 0.05, 0.5, 1, 1.5, and 2 mV were entered into PregCare, respectively. The output of PregCare was recorded and averaged over 3 repeated tests.

#### 3.1.2. Accuracy Evaluation

Relative error (RE) was used to evaluate the accuracy of PregCare. RE is the amplitude error between PregCare and the vital signs simulator, which was calculated as follows:

(1) $$\begin{array}{c} \text{RE}=\;\frac{A_{\text{t}}-A_{\text{e}}}{A_{\text{e}}}\times 100\%, \end{array}$$

where *A*_t_ is the signal amplitude recorded by PregCare and *A*_e_ is the signal amplitude from the vital signs simulator. RE less than 10% was acceptable in this study.

#### 3.1.3. Coefficient of Variation Evaluation

Coefficient of variation (CV) was used to evaluate the repeatability of multiple tests with PregCare, which was calculated as follows:

(2) $$\begin{array}{c} \text{CV}=\frac{\sigma }{\left|\mu \right|}\times 100\%,\\ \;\sigma =\sqrt{\frac{1}{N}\overset{N}{\underset{i=1}{\sum }}{\left(A_{\text{t}i}-\mu \right)}^{2}},\\ \mu =\frac{1}{N}\overset{N}{\underset{i=1}{\sum }}A_{\text{t}i}, \end{array}$$

where *A*_t*i*_ is the signal amplitude recorded by PregCare in the *i*^th^ test and *μ* is the averaged amplitude of *N* repeated tests and here *N* = 3. CV less than 1% was acceptable in this study.

#### 3.1.4. Bland–Altman Analysis

Bland and Altman analysis [18] has been utilized to assess the agreement between two measurement techniques. As a commercial instrument, PowerLab (ADInstruments Castle Hill, Australia) has been widely used in the physiological measurement. Therefore, Bland–Altman plots were applied to PregCare and PowerLab to evaluate their agreement. In the Bland–Altman analysis, the distribution of the measurements was expressed as the mean difference and standard deviation (SD) between PregCare and PowerLab. In addition, the 95% limits of agreement, which were defined as the mean difference ± 1.96SD, were determined to assess the agreement between the PregCare and PowerLab.

### 3.2. Evaluation of PregCare on Pregnant Women

#### 3.2.1. Subject

20 healthy pregnant women (33.2 ± 3.4 years old) with 38∼41 gestational weeks were recruited at Beijing Union Medical College Hospital in China. The measurement was performed according to the Declaration of Helsinki (1989) of the World Medical Association and approved by the Local Ethics Committee of Beijing Union Medical College Hospital. The pregnant women were asked to sign consent after being informed of the aims, potential benefits, and risks of the study.

#### 3.2.2. Signal Recording and Preprocessing

8-channel EHG and TOCO signals were recorded simultaneously with the sampling rate of 250 Hz, and the recording duration was approximately 30 min. UC was annotated by maternal perception using the mark button.

The recorded EHG signals were firstly preprocessed by a digital low-pass filter (0∼3 Hz) [19] and then by a median filter to remove the unwanted signals, including the baseline drift, power line, mother movement, and fetal/maternal ECG signals.

#### 3.2.3. EHG Feature Extraction

**Figure 5:**
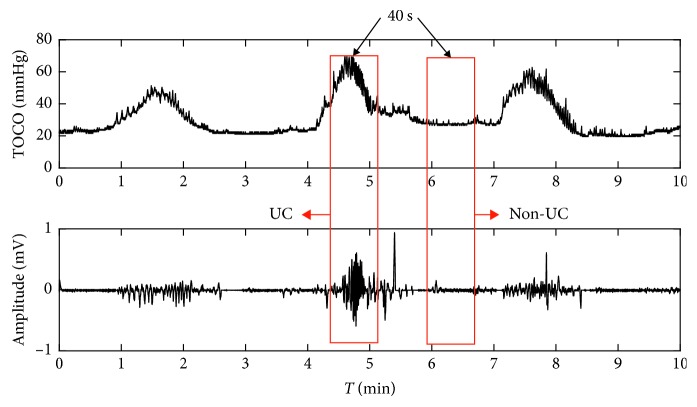
Example of the selection of UC and non-UC periods.

The UCs were determined and agreed to TOCO signals and the annotations made by the pregnant women. The EHG segment of 40 s duration corresponding to the UC was manually extracted from the EHG signal and confirmed by two experienced clinicians. Its corresponding non-UC period (40 s) was then extracted 40 s after the end of that UC.  Figure 5 gives one example of UC and non-UC periods from a subject. 40 EHG segments of UCs and 40 segments of non-UCs were obtained from the recruited pregnant women, respectively.

The power spectral density (PSD) of the EHG signal was depicted to indicate its power distribution with frequency. Referring to the previous studies [20–22], EHG features from time domain, frequency domain, and nonlinear domain including root mean square (RMS), peak frequency (PF), median frequency (MDF), and sample entropy (SamEn) were calculated from UC and non-UC, respectively. RMS is defined as the square root of the mean square of all sampling amplitude. PF corresponds to the largest amplitude peak as determined by the power spectrum. MDF is the frequency at which 50% of the total power within an epoch reaches. SamEn is used for assessing the complexity of physiological time-series signals. The feature values of 40 contractions and 40 noncontractions were extracted, respectively.

#### 3.2.4. Statistical Analysis

The means and SDs of EHG signal features were calculated for UCs and non-UCs. One-way ANOVA was employed using software SPSS 24 (SPSS Inc.) to assess the feature difference between UCs and non-UCs.

## 4. Results

### 4.1. PregCare Results

**Figure 6:**
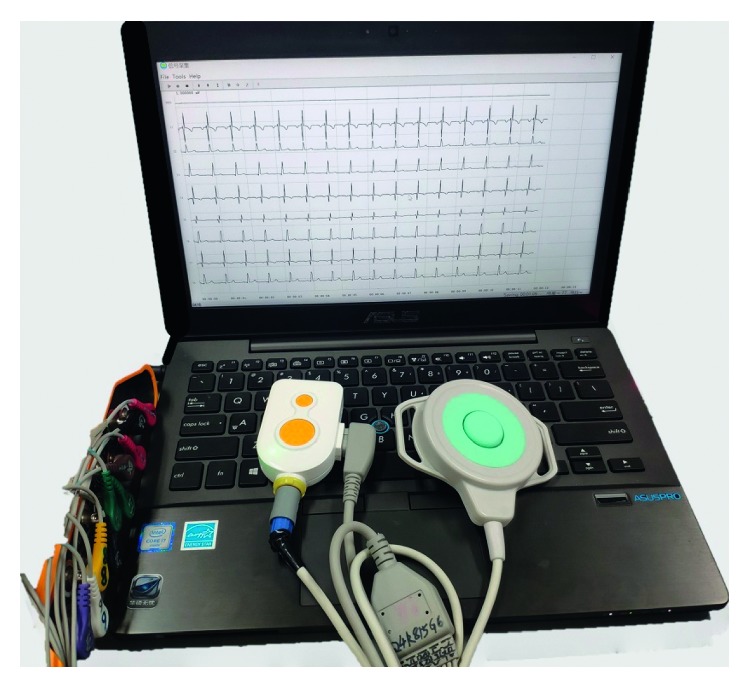
EHG recording system.

PregCare appearance and the recorded EHG and TOCO signals are shown in Figure 6.

The technical specifications of PregCare have been tested as follows: CMRR: 110 dB; bandwidth: 0–65 Hz; noise: 1 *μ*V; sample rate: 250 Hz; EHG signal resolution: 24 bit ADC; TOCO signal resolution: 12 bit ADC; transmission mode: BLE; effective transmission distance: 10 m in the absence of a barrier; power supply: 3.7 V Li battery; effective working time: >24 h; power consumption: 20 mW.

### 4.2. Evaluation Results with the Signal Simulator

Table 1 shows the evaluation results of PregCare. Both RE and CV were acceptable, which indicated that PregCare had high accuracy and repeatability.

**Figure 7:**
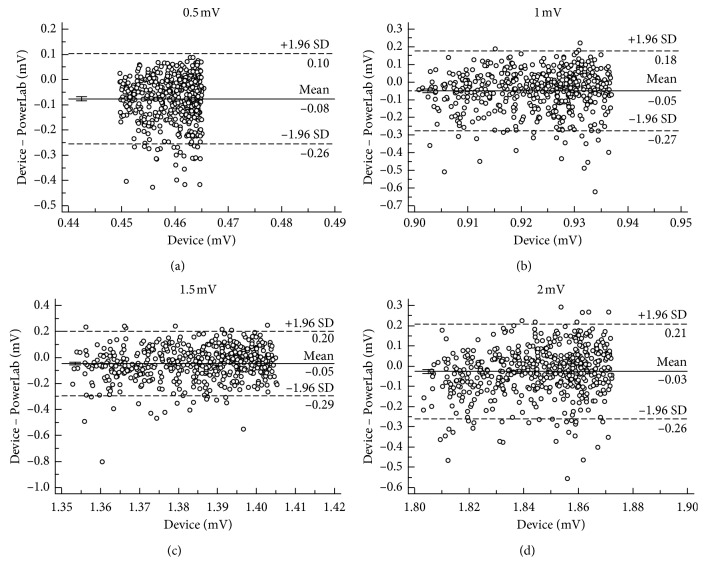
Bland–Altman plots of different signal amplitudes between PregCare and PowerLab: (a) *A*_e_ = 0.5 mV; (b) *A*_e_ = 1 mV; (c) *A*_e_ = 1.5 mV; (d) *A*_e_ = 2 mV.

Figure 7 shows the mean difference and 95% limits of agreement between PregCare and PowerLab. The small differences of mean and SD indicate the high agreement between PregCare and PowerLab.

### 4.3. Evaluation Results on Pregnant Women

**Figure 8:**
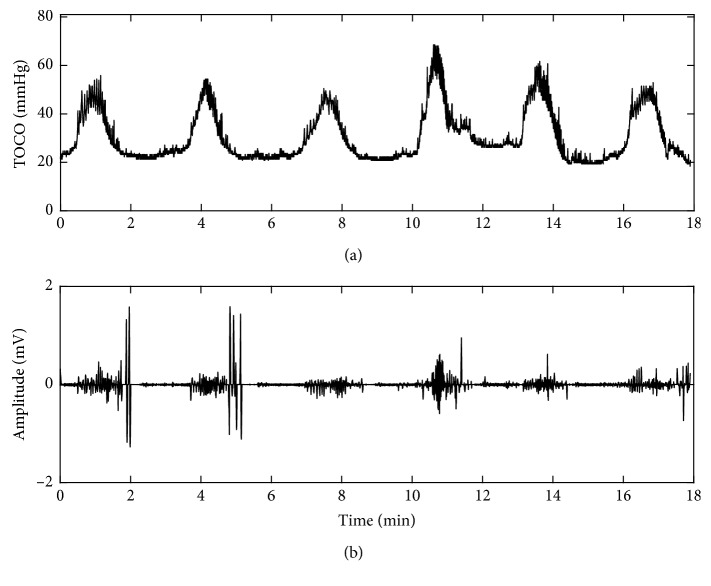
(a) TOCO and (b) EHG signals from a pregnant woman in term labor.

**Figure 9:**
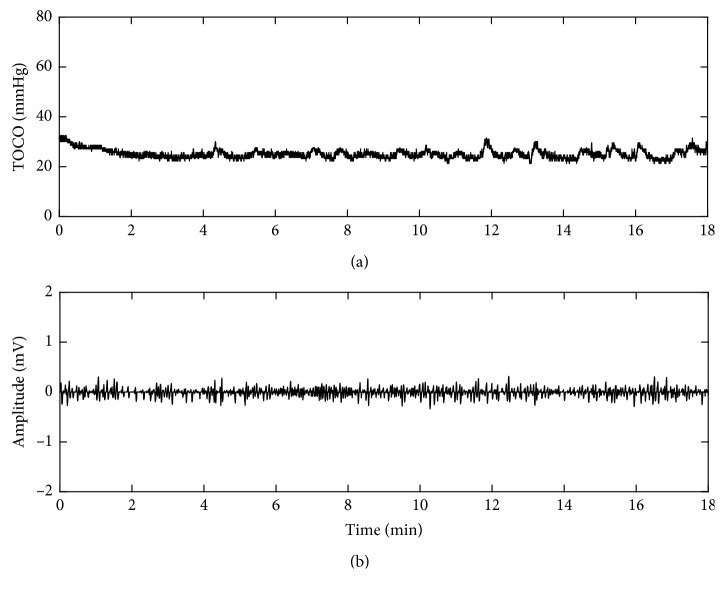
(a) TOCO and (b) EHG signals from a pregnant woman in nonlabor.

Figure 8 shows the TOCO signal and one channel preprocessed EHG signal from a pregnant woman in term labor who felt obvious UCs. The EHG bursts corresponding to UCs indicated by TOCO were distinctive. The duration and frequency of EHG bursts were consistent with the clinical experience about UCs.  Figure 9 shows TOCO and EHG signals collected from a pregnant woman in nonlabor who did not feel any UC during recording. Therefore, no burst was observed in the EHG signals.

### 4.4. Comparison of EHG Features between UCs and Non-UCs

**Figure 10:**
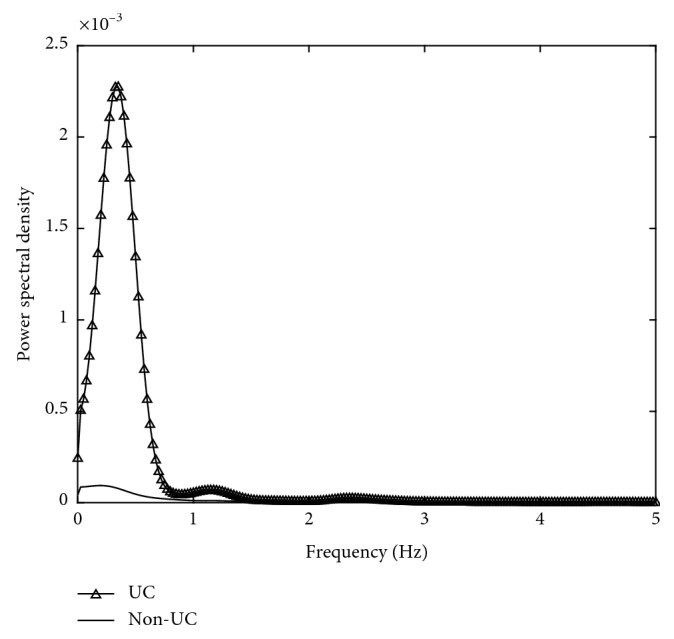
PSD of UCs and non-UCs.

Figure 10 shows the PSD of EHG during UCs and non-UCs. It was observed that EHG energy was predominantly in the 0–0.7 Hz frequency band. The power of UC was much larger than non-UC.

EHG features were compared between UCs and non-UCs, as shown in Table 2. Table 2 indicates RMS of EHG from UCs was significantly larger than non-UCs (*p* < 0.05). PF and SamEn of EHG from UCs were significantly smaller than non-UCs (*p* < 0.05). MDF was not significantly different between UCs and non-UCs (*p* > 0.05).

### 4.5. Comparison with Existing Devices

The existing devices such as Bloomlife and Monica have different application purposes. Bloomlife provides frequency, duration, and pattern of UC, and Monica performs the complex calculations to extract the fetal/maternal heart rate and UC waveform. Both of them only offer one channel of the overall UC. Our system, PregCare, can provide the local uterine activities with 8 electrodes.

Comparison with the commercial devices including Bloomlife and Monica is shown in Table 3.

## 5. Discussion

It has been shown that EHG is able to provide valuable information about the changes in the electrical properties of the myometrium, and EHG-derived UCs are more likely to be adequate and are more easily assessed than TOCO contraction patterns [25]. Our developed EHG recording system could be used to monitor UCs for a long time and predict preterm labor further.

The EHG recording system, PregCare, was specially designed for scientific research purpose, which collected EHG, TOCO, and maternal perception simultaneously. It could provide the comprehensive information of uterine activities with 8 electrodes covering the fundus, body, and cervix of the uterus. Therefore, the propagation characteristics of UC including its direction and velocity can be obtained to assist in prediction of imminent delivery and diagnosis of preterm labor [13, 14]. Moreover, PregCare also provides TOCO and maternal perception as references for UC recognition with EHG signals. PregCare has the advantages of being portable, light, and low cost with wireless transmission, which allows it to be used both at clinics and home for long-term monitoring of UCs. PregCare has been evaluated to have high accuracy and repeatability and good agreement with the commercial device PowerLab.

With our developed recording system, the UCs could be recognized more accurately combining EHG, TOCO, and maternal perception. EHG bursts corresponding to UCs in term labor were very obvious compared with nonlabor. Referring to TOCO and maternal perception, EHG segments corresponding to UCs could be recognized more accurately, which will provide support for automatic detection of UCs and prediction of preterm labor with EHG signals further. Therefore, our developed EHG recording system has a promising prospect of application.

The EHG signal within 0–0.7 Hz which is related to the propagation of uterine activity has been used to reflect the UC coordination in labor [26]. RMS during UC was distinctively larger than non-UC which was consistent with the previous work [5], in which the EHG signal amplitude increased as a result of increased myometrial activity as delivery approached. PF of UC was significantly smaller than non-UC, which had been considered as the most predictive of true labor [3]. SamEn measures the irregularity of a time series of finite lengths. The more unpredictable the time series is, the higher its sample entropy is. The decreased SamEn in UC suggested the EHG signal became more regular than that during non-UC. Especially for EHG signals from the imminent delivery, their UCs became more coordinated than non-UC, which also conformed to clinical experience. SamEn has also been a significant feature for distinguishing between term and preterm delivery [6].

The paper focused on the research and development of the EHG recording system. Therefore, only 20 pregnant women were selected for preliminary analysis and evaluation of the performance of PregCare. More data will be taken into account for robust analysis in further study. The results obtained with the conventional analysis methods demonstrated that PregCare could also indicate the EHG features as reported in the published papers [21, 22]. Currently, EHG signals were segmented into UCs and non-UCs manually by the investigators offline. More efforts will be made to recognize UC automatically and extract UC features including intensity, duration, and frequency in real time, which has a promising prospect in pregnancy care.

## 6. Conclusions

In conclusion, the study has developed a low-cost and portable EHG recording system which can record EHG signals reliably. It can be used for long-term monitoring of UCs and prediction of preterm delivery.

## Figures and Tables

**Table 1:** Evaluation of the PregCare.

Group	1	2	3	4	5
*A*_e_ (mV)	0.05	0.5	1	1.5	2
*A*_t_ (mV)	0.046	0.460	0.923	1.385	1.846
RE (%)	8	8	7.6	7.7	7.7
CV (%)	0.022	0.013	0.011	0.022	0.014

**Table 2:** Comparison of EHG features between UCs and non-UCs in mean ± SD.

Feature	Non-UC	UC
RMS (mV)	0.038 ± 0.036	0.075 ± 0.063^*∗*^
PF (Hz)	0.34 ± 0.0390	0.27 ± 1.004^*∗*^
MDF (Hz)	0.5703 ± 0.17	0.5606 ± 0.25
SamEn	0.0647 ± 0.031	0.0430 ± 0.019^*∗*^

^*∗*^ *p* < 0.05 between UCs and non-UCs.

**Table 3:** Comparison with similar devices.

	Bloomlife [23]	Monica [24]	PregCare
Channel number	2	3	9
Signal measured	Abdominal electrophysiological signal	Abdominal electrophysiological signal	Abdominal electrophysiological signal and strain signal
Data storage	Wirelessly transmitted to a mobile phone	Stored in the internal micro-SD card or wirelessly transmitted to a computer	Wirelessly transmitted to a computer
Application purpose	Frequency, duration, and patterns of UC	Fetal/maternal heart rate and UC	UC monitoring and preterm labor prediction

## Data Availability

The data used to support the findings of this study are available from the corresponding author upon request.
